# Open Surgical Versus Endovascular Repair for Type B Aortic Dissection: Umbrella Review and Meta-Analysis

**DOI:** 10.1177/00033197251392660

**Published:** 2025-12-12

**Authors:** Hassan Al-Thani, Osamah Alrawi, Eman Elmenyar, Mashhood Naduvilekandy, Shams O. Alkhateeb, Waqar Mogassabi, Lama Alkahlout, Ayman El-Menyar

**Affiliations:** 1 Department of Surgery, Trauma, and Vascular Surgery, Hamad Medical Corporation, Doha, Qatar; 2 Department of Anesthesiology, Hamad Medical Corporation, Doha, Qatar; 3 Faculty of Medicine, Bahçeşehir University, Istanbul, Turkey; 4 Department of Trauma & Vascular Surgery, Clinical Research, Hamad Medical Corporation, Doha, Qatar; 5 Department of Medical Education, Hamad Medical Corporation, Doha, Qatar; 6 College of Medicine, Qatar University, Doha, Qatar; 7 Department of Clinical Medicine, Weill Cornell Medical College, Doha, Qatar

**Keywords:** type B, aortic dissection, endovascular, open surgery, mortality

## Abstract

This umbrella review (UR) synthesizes the current evidence comparing thoracic endovascular aortic repair (TEVAR) with open surgical repair (OSR) for the management of type B aortic dissection (TBAD), with a focus on both early and long-term outcomes. A systematic literature search was performed using PubMed, Embase, Cochrane Library, and Web of Science (2014-2025). Eleven systematic reviews met the inclusion criteria. Short-term mortality was lower with TEVAR, ranging from 2% to13.4%, while it was 4.5% to 19% with OSR. The meta-analysis showed a risk ratio (RR) of 0.51 (95% CI: 0.43-0.59; *I*^2^ = 64.6%) in favor of TEVAR. Long-term survival was comparable. TEVAR was associated with fewer complications, including cardiac events (odds ratio [OR]: 0.42-0.79), pulmonary events (OR: 0.51-0.57), renal failure (OR: 0.53-0.63), and bleeding (OR: 0.24 and RR: 0.44). For stroke, the UR showed mixed results (OR: 0.23-1.11), but the meta-analysis showed a lower risk with TEVAR (RR: 0.67; 95% CI: 0.54-0.82; *I*^2^ = 8.7%). Paraplegia rates were comparable (RR: 0.88; 95% CI: 0.53-1.47; *I*^2^ = 22.7%). Despite the observed moderate to high heterogeneity among most studies (*I*^2^ = 30%-64.6%), the overall trend favored TEVAR in terms of early outcomes. There was a need for further high-quality, longitudinal studies and randomized controlled trials.

## Introduction

Aortic dissection is a critical vascular condition characterized by a tear in the aortic wall, leading to the formation of a false lumen.^1^ The condition is classified based on the anatomical location and extent of the dissection. The DeBakey classification categorizes dissections into 3 types: type I involves the ascending aorta and may extend to the descending aorta; type II is confined to the ascending aorta; and type III only affects the descending aorta.^2^ The Stanford classification simplifies this into type A, which involves the ascending aorta, and type B, which is confined to the descending aorta, beyond the left subclavian artery.^2^

Type B aortic dissection (TBAD), presents unique management challenges. Historically, open surgical repair (OSR) has been the conventional treatment, aiming to directly repair the aortic wall and address related complications.^3^ Although effective, this approach is invasive and carries significant operative risks and prolonged recovery times. Depending on the clinical features, progression of the dissection, and patient’s overall condition, TBAD could also be managed conservatively where the dissection is stable, and complications are not imminent.^3,4^

The development of endovascular repair techniques has introduced a less invasive alternative.^3^ Endovascular repair involves deploying a stent-graft within the aorta to stabilize the vessel and prevent further dissection, potentially reducing the risks associated with traditional surgery.^5^ However, the applicability of thoracic endovascular aortic repair (TEVAR) remains dependent on anatomical and patient-specific factors. Not all patients with TBAD are suitable candidates for endovascular repair due to factors such as unfavorable vascular anatomy, inadequate proximal or distal landing zones, and connective tissue disorders that predispose them to long-term complications.^6,7^ Additionally, concerns about stent-graft durability, endoleak development, and the potential need for reintervention emphasize the importance of long-term surveillance after TEVAR.^8^ These factors highlight the need for a precise approach to treatment selection, balancing the benefits of reduced early morbidity with the risks of later complications.

While some evidence suggests that TEVAR may offer advantages in terms of reduced early mortality and postoperative complications, conflicting data exists regarding its long-term efficacy compared with OSR. The comparative effectiveness of these 2 treatment strategies (TEVAR vs OSR) has been the subject of numerous systematic reviews and meta-analysis. However, findings from these studies are often inconsistent due to variations in study design, patient demographics, and follow-up periods. An umbrella review (UR) synthesizes evidence from systematic reviews (SRs), providing a comprehensive understanding of the relative benefits and risks of open versus endovascular repair for TBAD. By pooling data from multiple systematic reviews, the present UR provides a robust comparison, focusing on both early/short and long-term outcomes.

## Methods

### Data Sources and Search Strategy

A comprehensive search was conducted using PubMed, Embase, Cochrane Library, and Web of Science to identify systematic reviews and meta-analyses published between January 2014 and February 2025. The search strategy included keywords and Medical Subject Headings (MeSH) terms such as “Type B aortic dissection,” “thoracic aorta,” “open repair,” “endovascular repair,” “aortic surgery,” “stenting,” “repair,” and “aorta,” combined with “systematic review” OR “meta-analysis” to specifically retrieve relevant studies. Additionally, reference lists of included articles were manually screened by 3 independent reviewers to identify any further eligible systematic reviews or meta-analyses and any discrepancies were resolved through discussion or with the senior author. The selection process was documented using the Preferred Reporting Items for Systematic reviews and Meta-Analyses (PRISMA) 2020 flow diagram (Figure 1).^9^ The review protocol was registered with The International Prospective Register of Systematic Reviews (PROSPERO): CRD42024593227 on October 3, 2024 (Supplemental Material).^10^

**Figure 1.**
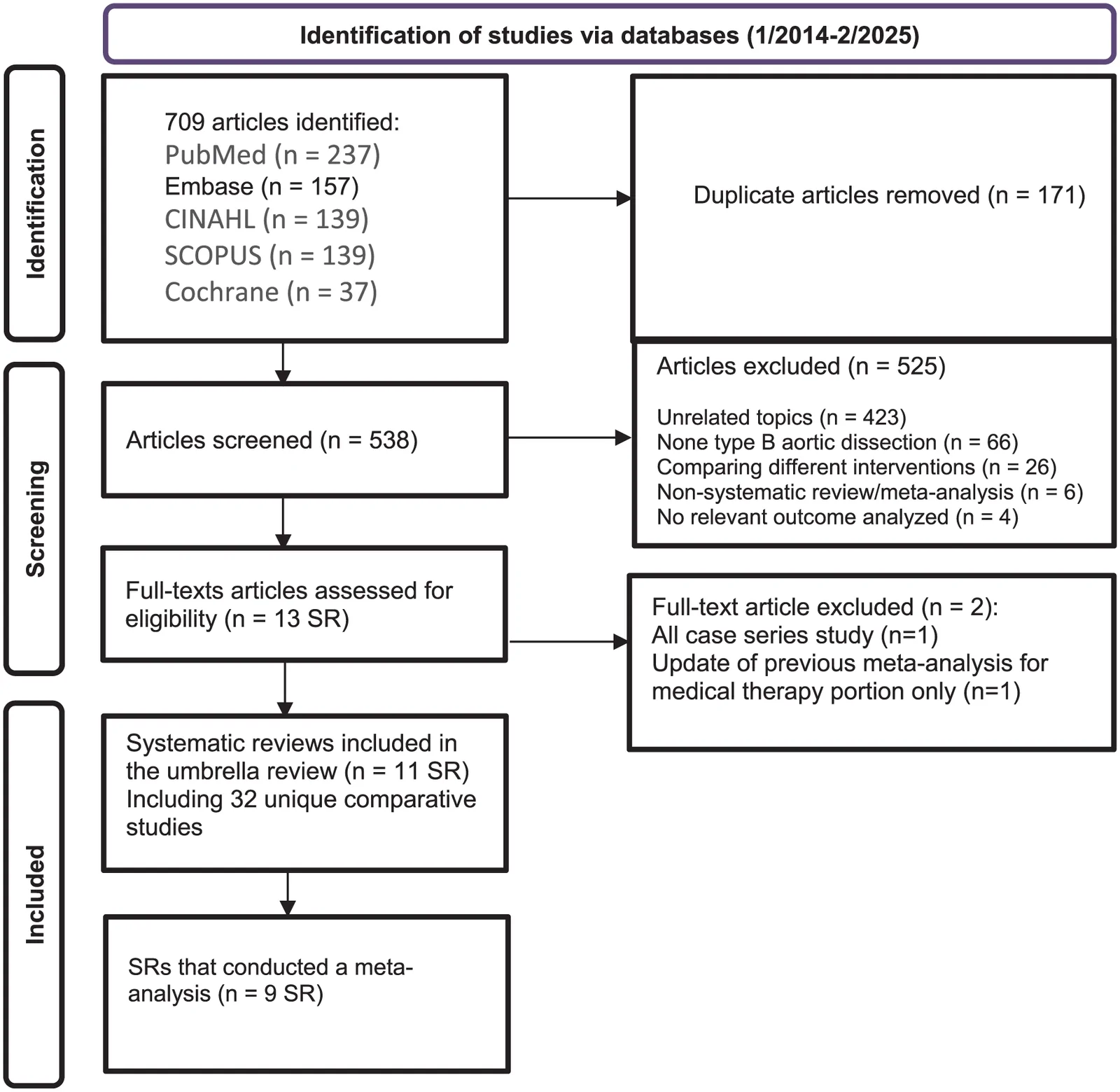
PRISMA flow chart for the included systematic review and meta-analysis.

### Inclusion and Exclusion Criteria

*Population/Intervention/Comparison/Outcome (PICO). P*: patients with TBAD; *I*: non-medical treatment; *C*: OSR or TEVAR; *O*: early/short (in-hospital or 30-day) and long-term outcomes (1, 3, and 5 years). Studies were included if they were systematic reviews or meta-analyses comparing OSR and TEVAR in adult patients and reported outcomes such as mortality, operative complications, reinterventions, or comorbidities, including hypertension, diabetes mellitus, and chronic kidney disease. Studies were excluded if they focused on pediatric or non-human populations, employed study designs not aligned with the inclusion criteria (such as narrative reviews or editorials), did not provide comparative analyses, or assessed outcomes beyond the intended scope. Non-English articles were not included.

### Data Extraction

Data extraction was conducted in 2 stages by at least 2 independent reviewers to ensure accuracy. In the first stage, we extracted study-level information from each systematic review, including author, year, study duration, number and type of included studies, population characteristics, age, sex, operative complications, and mortality data. In the second stage, we collected outcome-specific data—such as event counts and total sample sizes for OSR and TEVAR arms—from the individual studies included within these reviews. Any discrepancies were resolved through discussion or with a third reviewer. Overlapping or duplicate data were identified and excluded to avoid bias, and all extracted data were compiled and cross-verified for consistency.

### Methodological Quality and Certainty of Evidence

The quality of the SRs included was assessed using A MeaSurement Tool to Assess systematic Reviews (AMSTAR 2), a validated tool designed to critically appraise the methodological rigor of SRs.^11^ Each study was evaluated across 16 domains, including protocol registration, risk of bias assessment, and data synthesis methods. The Grading of Recommendations, Assessment, Development and Evaluations (GRADE) tool was used to analyze the certainty of evidence for the meta-analysis outcomes.^12^

### Meta-Analysis

After identifying individual studies from the included SRs, meta-analyses were conducted using random-effects models (DerSimonian-Laird method) to account for between-study heterogeneity. This model assumes that the true effect size may vary between studies, and it incorporates both within-study sampling error and between-study variability into the pooled estimate. Heterogeneity was quantified using the *I*^2^ statistic and τ^2^. *I*^2^ statistic quantifies the proportion of total variation in study results, that is, due to true differences between studies (heterogeneity) rather than just chance, providing a percentage from 0% to 100%. The tau-squared (τ^2^) statistic estimates the actual variance of these true effects between studies within a random-effects model, with a high value indicating substantial variation in true effects across studies.^13^

A single-arm meta-analysis was performed using the *metaprop()* function from the meta package to estimate the pooled short-term mortality rate (in-hospital or 30-day) in patients who underwent OSR or TEVAR.

To compare outcomes between interventions, risk ratios (RR) with 95% confidence intervals (CI) were adopted for short-term mortality, paraplegia, and stroke, comparing TEVAR versus OSR. However, the odds ratio and relative risk were reported in some SRs. These comparative analyses were conducted using the *metabin()* function, applying the inverse variance method with the DerSimonian-Laird estimator for between-study variance (method.tau = “DL”).

#### Publication Bias

Methods for detecting publication bias included visual inspection of funnel plots and statistical tests like Egger’s regression test.^14^ A *P* < .05 indicates asymmetry in the funnel plot and the presence of publication bias or small-study effects. To mitigate publication bias such as overestimation of effect sizes, false-positive results or distorted summary of evidence, sensitivity analyses were performed. All analyses were conducted in RStudio (version 2024.10.31; Posit Software, PBC, Boston, MA, USA) using R (version 4.4.2; R Foundation for Statistical Computing, Vienna, Austria) with the meta package (version 8.0.2; Schwarzer, Carpenter & Rücker).

## Results

### Characteristics of Included SRs (UR)

The comprehensive literature search identified 11 SRs^15-25^ and 9 of them were used for meta-analyses. This UR consolidates the evidence from these SRs and Meta-analysis to evaluate the efficacy and outcomes of TEVAR compared with OSR in the management of TBAD. The SRs and meta-analysis were published between January 2014 and February 2025. Some of the underlying individual reviews, however, extended their literature searches from as early as 1980, or from database inception up to 2023.^16,23^ These SRs and meta-analysis themselves encompassed a large number of primary research articles. For example, the number of primary studies included in these reviews ranged from 9 to 49 articles for comparative meta-analyses, and up to 39 or 54 studies in comprehensive reviews (Table 1).^15,16,18,22^

**Table 1.** Characteristics of Included SRs.

Author/year/duration	Type and no of articles	Total population		Mean (SD)/median (IQR) age^a^		Male	
		OSR	TEVAR	OSR	TEVAR	OSR	TEVAR
Harky et al (2019)^15^ (2009-2015)	2 Prospective 7 Retrospective	11 618	6575	61.3 (9.3)	66.6 (12.5)	59.3%	59.9%
Ahmed et al (2024)^16^ (1990-2022)	3 RCTs 46 Cohort	34 265	20 953	64.6 (7.4)	64.0 (5.0)	72%	70.1%
Donghai Liu et al (2020)^17^ (2005-2016)	18 Observational	1546	2860	NA	NA	NA	NA
Boufi et al (2019)^18^ (2000-2017)	9 Prospective 30 Retrospective	1079	1271	58.1 (0.9)	59 (0.9)	NA	NA
Li et al (2018)^19^ (no duration)	16 Retrospective cohort	125	1554	NA	NA	NA	NA
Lombardi et al (2023)^20^ (2000-2021)	Case series: 8 Case reports: 24	22	38	49.1 (8.7)	57.8 (9.9)	90.9	76.3
Zhu et al (2016)^21^ (2004-2015)	1 Prospective 8 Retrospective	303	436	NA	NA	NA	NA
Moulakakis et al (2014)^22^ (2006-2013)	54 Observational	1276	2531	NA	NA	NA	NA
Chandra et al (2022)^23^ (1980-2020)	10 Retrospective 27 Case reports	35	112	57 (48-85)^a^	53.5 (46-63)^a^	80%	78.5%
Jianping Liu et al (2019)^24^ (2006-2018)	18 Observational	6420	3244	NA	NA	NA	NA
Ying Yu et al (2023)^25^ (2010-2015)	11 Retrospective	3667		61.2		60.6	

Abbreviations: NA, not available; TEVAR, thoracic endovascular aortic repair; OSR, open surgical repair; IQR, interquartile range; SR, systematic reviews.

The methodological nature of the primary studies included in these reviews predominantly comprised observational designs, with some analyses incorporating randomized controlled trials (RCTs).^15-17,19^ However, more recent assessments such as Yu et al continued to rely exclusively on retrospective observational studies despite being framed as controlled comparisons.^25^ Certain specialized SRs also incorporated case series and case reports, especially when addressing rare clinical presentations or complications like mesenteric malperfusion, where no RCTs were found.^20,23^

The primary comparisons across these numerous analyses consistently focused on the efficacy and outcomes of TEVAR and OSR. Several reviews directly compared all 3 treatment modalities.^16,17,20,22^ Other systematic reviews and meta-analyses concentrated on specific comparisons, such as TEVAR versus OSR for chronic TBAD, acute TBAD, and endovascular versus surgical management for TBAD complicated by mesenteric malperfusion.^15,18,23^ Lombardi et al focused on open, hybrid, and total endovascular approaches for TBAD in the presence of an aberrant right subclavian artery (ARSA), also without a medical treatment comparison arm.^20^

The size of the patient cohort varied widely across the included studies, ranging from a few dozen to several tens of thousands. While some meta-analyses and SRs pooled large datasets from multiple centers, others were based on smaller or more focused cohorts. This variation reflects the differing scopes and methodologies of the studies but provides a comprehensive overview across diverse populations and settings.

Patient demographics varied across individual studies and cohorts but generally indicated a patient population with a mean age typically ranging from the mid-50s to mid-60s with a consistent male predominance, often around 60% to 90% of the cohort in both groups.^15,18,20,23,25^

## Outcomes

### In-Hospital/30-Day Mortality

Across multiple studies, TEVAR showed generally lower mortality rates compared with OSR in patients with TBAD. Mortality for TEVAR ranged from 2% to13.4%, while OSR mortality ranged from 4.5% to19% (the mortality values were collected from different SRs, not from a single pooled analysis, therefore, a *P* value cannot be meaningfully provided),^15,18,20,23^ indicating a trend toward better short-term survival with TEVAR with several studies highlighting statistically significant differences (Table A1).

Pooled effect estimates showed the superiority of TEVAR. Meta-analyses have reported OR ranging from 0.24 to 0.54, with (*I*^2^ = 30%-33%) indicating a 46% to 76% relative reduction in early mortality risk with TEVAR compared with OSR.^17,18^ Relative risk estimates (eg, RR: 0.59 from Yu et al^25^) further support TEVAR’s survival benefit. These findings are consistent across both acute and chronic TBAD subtypes such as those undergoing repair for complicated dissections or conditions like ARSA involvement.^15,18,20,25^

The meta-analysis conducted using the original studies included in the SRs showed that TEVAR had a pooled proportion of 4% mortality from 21 709 patients and OSR had a 17% mortality rate from 32 438 patients. The risk ratio calculated from the 31 comparative studies was found to be 0.51 (95% CI: 0.43-0.59; *I*^2^ = 64.6%) significantly reducing the short-term mortality in the TEVAR group (Figure 2). However, the funnel plot and Egger’s test (*P* = .003) showed small studies with opposing results may be missing or introducing publication bias (Figure 3). Subgroup analysis demonstrated a consistent survival benefit for TEVAR in both acute and chronic cohorts, though the chronic subgroup was represented by far fewer studies and patients, limiting the certainty of conclusions in this population (Figure 4). Leave-one-out sensitivity analysis and line diagram showed that no single study significantly altered the pooled effect estimate, and heterogeneity remained broadly stable (*I*^2^ ~64%-66%), except when omitting the Treffalls et al study,^26^ which reduced *I*^2^ to 48.6% (Figure B1). Another sensitivity analysis to avoid the publication bias was performed by removing studies with sample size <150 patients. The remaining studies (n = 13) gave RR 0.53 (95% CI: 0.44-0.65; *I*^2^ = 81.3%) and a non-significant Egger’s test value (*P* > .05; Figures B2 and B3). Therefore, even after sensitivity analysis the RR did not substantially change.

**Figure 2.**
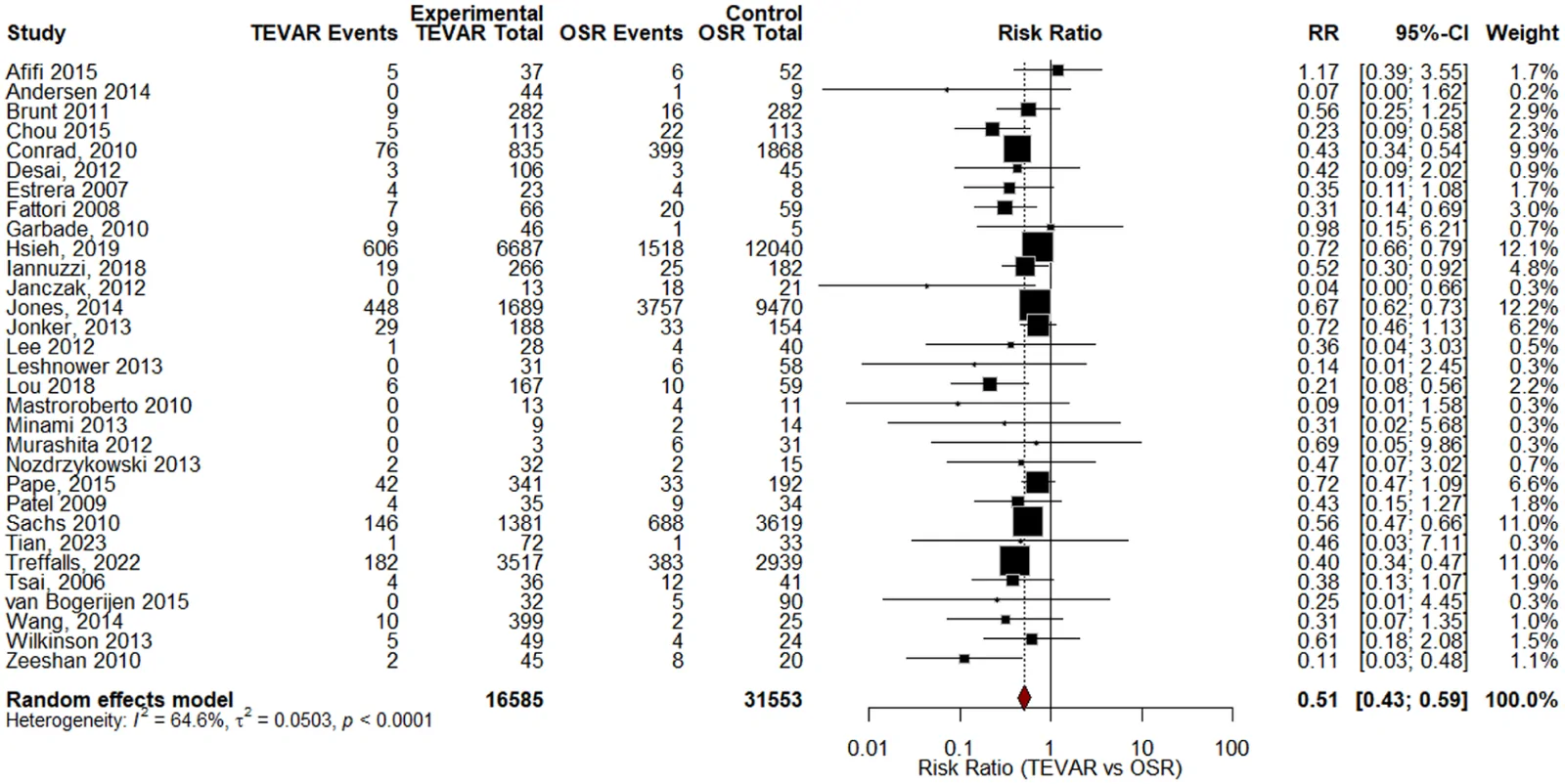
Forest plot for the short-term mortality (in hospital/30 days).

**Figure 3.**
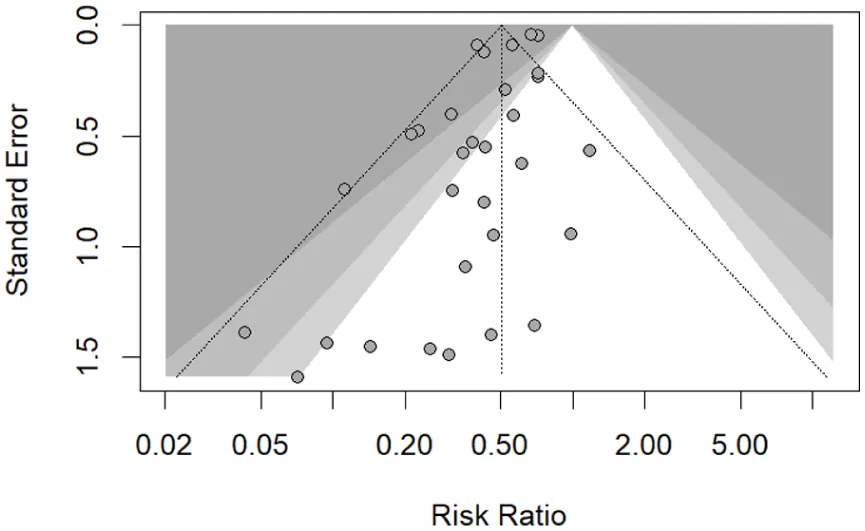
Funnel of short-term mortality studies.

**Figure 4.**
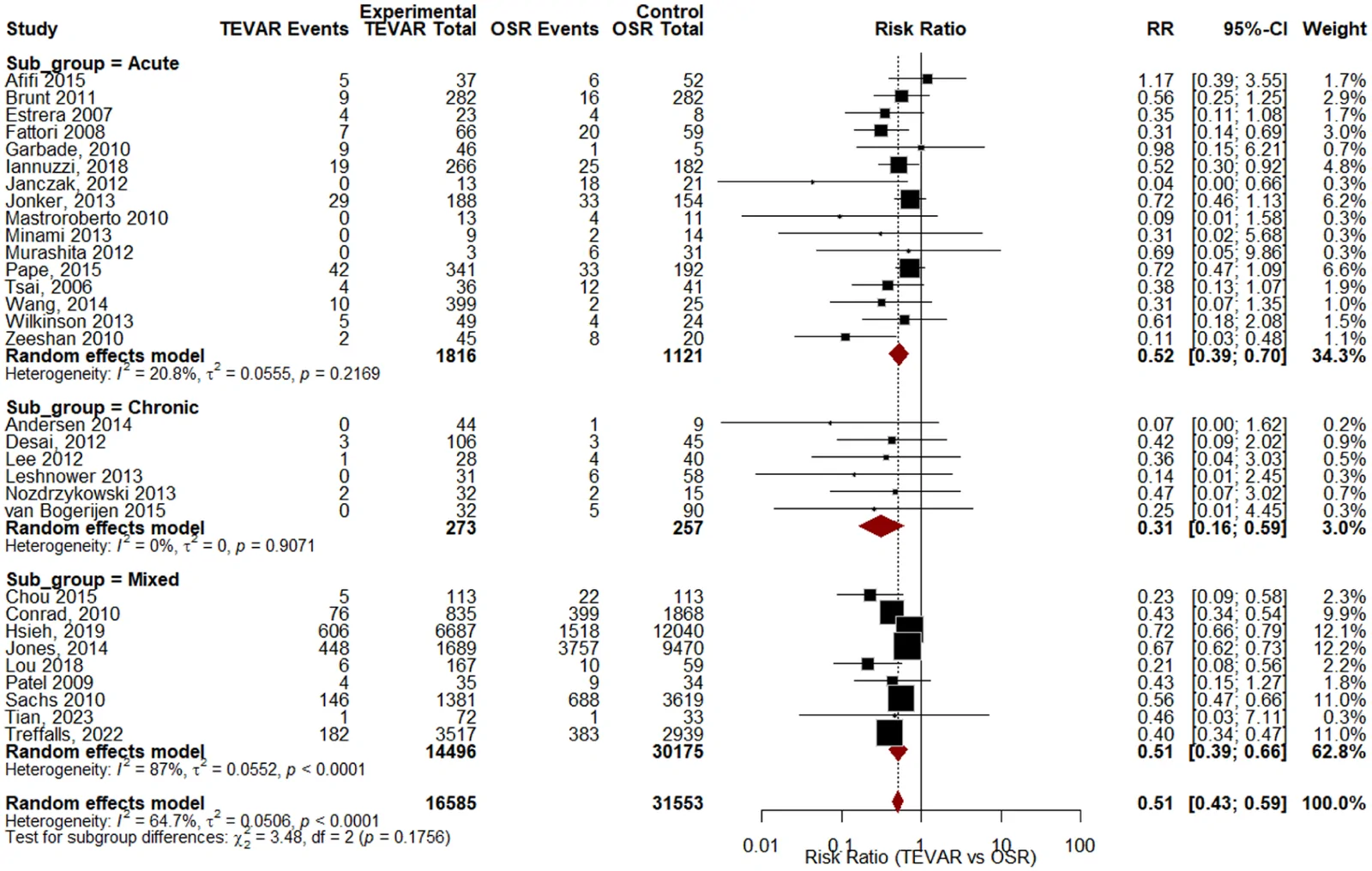
Subgroup analysis for survival benefit for TEVAR in both acute and chronic cohorts.

### Long-Term Survival

While TEVAR often shows early survival benefits over OSR, long-term outcomes appear largely comparable. Harky et al^15^ reported higher 1-year all-cause mortality for OSR (24.3%) versus TEVAR (14.3%), but similar mortality at 5 years (OSR: 46.7% vs TEVAR: 49.7%). Boufi et al^18^ reported no significant differences in survival at 1 year (OR: 0.73; 95% CI: 0.34-1.55; *P* = .41) or 3 years (OR: 1.19; 95% CI: 0.42-3.32; *P* = .73). Dongai Liu et al^17^ similarly found no significant differences at 1 year (OR: 0.41; 95% CI: 0.11-1.63; *P* = .21), 3 years (OR: 0.65; 95% CI: 0.22-1.87; *P* = .42), or ≥5 years (OR: 1.27; 95% CI: 0.32-5.01; *P* = .73). Moulakakis et al^22^ reported 1-year survival rates ranging from 62% to 100% (TEVAR) and 74.1% to 86.0% (OSR), and 5-year survival rates from 61% to 87% (TEVAR) and 44% to 82.6% (OSR). Li et al^19^ found a pooled hazard ratio for long-term survival of 0.88 (95% CI: 0.54-1.44), indicating no significant difference.

### Cardiac Complications

TEVAR was generally associated with fewer cardiac complications than OSR (Table A2). Ahmed et al^16^ reported a lower mean absolute risk for cardiac complications with TEVAR (0.104 ± 0.039) compared with OSR (0.184 ± 0.051), although contrast plots showed no statistically significant difference. Donghai Liu et al^17^ found a significant reduction in cardiac complications with TEVAR versus OSR (OR: 0.42; 95% CI: 0.33-0.54; *P* < .0001). Similarly, Harky et al^15^ reported a higher in-hospital cardiac complication rate for OSR (8.9%) compared with TEVAR (5.0%; *P* = .002). However, Jianping Liu et al,^24^ focusing specifically on myocardial infarction as a subset of cardiac complications, found no significant difference between TEVAR and OSR (OR: 0.79; 95% CI: 0.29-2.16; *P* = .650), with no evidence of heterogeneity.^24^

### Pulmonary Complications

TEVAR consistently demonstrated a lower risk of pulmonary complications compared with OSR. The meta-analysis by Donghai Liu et al^17^ found that TEVAR significantly reduced the incidence of pulmonary complications versus OSR, with an OR of 0.57 (95% CI: 0.48-0.68; *P* < .0001). Jianping Liu et al^24^ similarly reported a significantly reduced risk of respiratory failure in the TEVAR group (OR: 0.51; 95% CI: 0.32-0.82; *P* = .006) compared with OSR. Ying Yu et al^25^ also found that TEVAR had a lower risk of respiratory complications (RR: 0.67; 95% CI: 0.60-0.76; *P* < .00001) when compared with OSR. Providing more granular data, Boufi et al^18^ reported that respiratory complications occurred in 24.9% of OSR cases, compared with only 4% after TEVAR with OSR patients being 6.88 times more likely to develop severe respiratory complications (OR: 6.88; 95% CI: 1.52-31.02; *P* = .01).

### Renal Complications (Renal Failure or Dialysis)

TEVAR was generally associated with a lower risk of renal complications compared with OSR. Ahmed et al^16^ reported the lowest mean absolute risk of renal failure with TEVAR (mean: 0.119 ± 0.022), while OSR had the highest (mean: 0.200 ± 0.027), with the difference between the 2 being statistically significant (OR: 1.87 [1.19-2.89]). Harky et al^15^ also observed a significantly higher rate of renal failure requiring hemofiltration or dialysis in OSR (25.4%) compared with TEVAR (16.6%; *P* = .006). Ying Yu et al^25^ found a reduced risk of dialysis with TEVAR (RR: 0.55; 95% CI: 0.47-0.65; *P* < .0001). Likewise, Jianping Liu et al^24^ reported a significantly lower risk of acute renal failure with TEVAR (OR: 0.53; 95% CI: 0.30-0.94; *P* < .01).

Boufi et al,^18^ in non-comparative pooled data, noted a dialysis requirement of 5.2% after OSR and 0% after TEVAR. However, their comparative meta-analysis did not show a statistically significant difference in the need for permanent dialysis (OR: 2.99; 95% CI: 0.56-15.97; *P* = .20), possibly reflecting differences in the study designs. Li et al^19^ found no significant difference in renal failure rates between TEVAR and OSR (OR: 0.63; 95% CI: 0.32-1.24), although the direction of effect numerically favored TEVAR similar to Donghai Liu et al (OR: 0.57; 95% CI: 0.30-1.06; *P* = .08).^17^

### Stroke

Comparing stroke risk between TEVAR and OSR showed mixed findings. Several studies suggested a trend toward lower stroke risk with TEVAR. Ying Yu et al^25^ reported a significantly reduced stroke risk with TEVAR compared with OSR (RR: 0.71; 95% CI: 0.51-0.98; *P* = .03). Similarly, Boufi et al^18^ found a significantly lower early stroke rate for TEVAR (OR: 0.23; 95% CI: 0.04-0.98; *P* = .04), with cumulative stroke rates of 2.7% for TEVAR and 4.5% for OSR. Moulakakis et al^22^ reported higher cerebrovascular event rates in the OSR group (6.8%) compared with TEVAR (3.9%). The comparative studies included in the SRs showed a reduced stroke rate in TEVAR group (RR: 0.67 95% CI 0.54-0.82; *I*^2^ = 8.7%; Figure 5).

**Figure 5.**
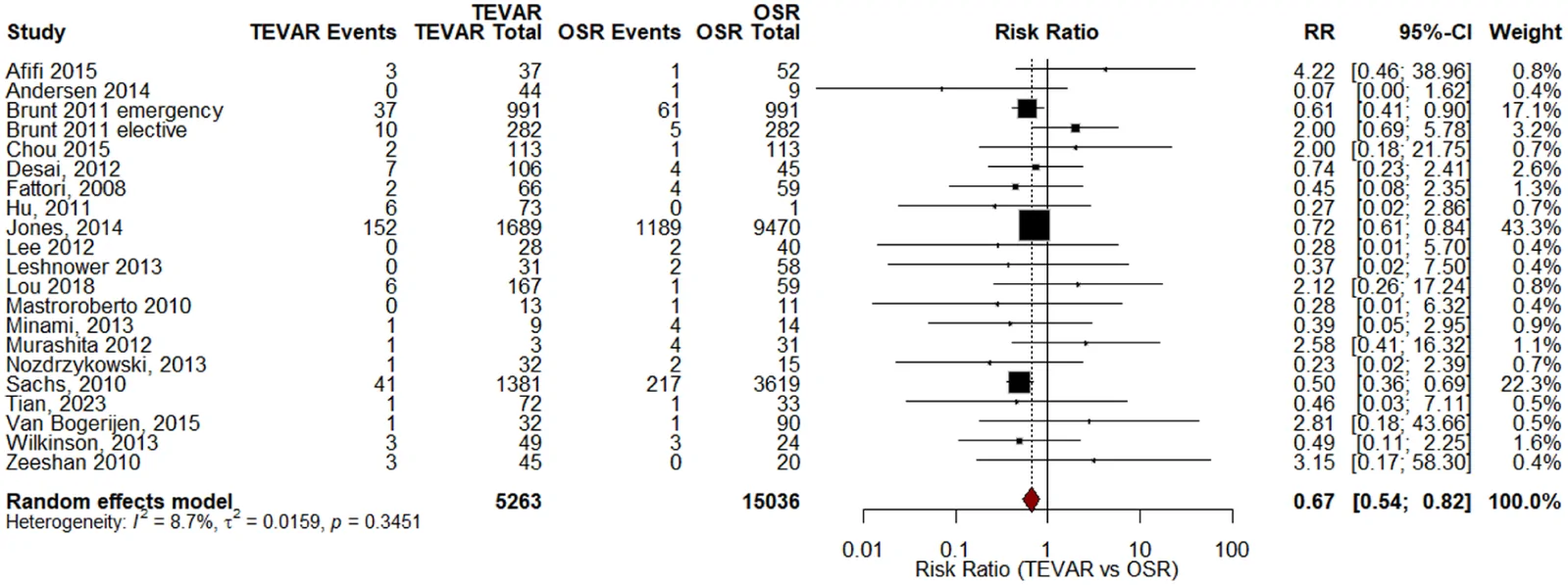
Forest plot for stroke studies.

However, several meta-analyses did not find statistically significant differences. Harky et al^15^ reported a slightly lower stroke rate in TEVAR (3.7%) versus OSR (4.7%) that was not statistically significant (*P* = .71). Donghai Liu et al^17^ also found no significant difference (OR: 1.11; 95% CI: 0.42-2.89; *P* = .84), a result echoed by Jianping Liu et al^24^ (OR: 0.98; 95% CI:0.48-1.97; *P* = .947), and Li et al^19^ (OR: 1.10; 95% CI: 0.53-2.28).

The funnel plot revealed no evidence of publication bias, which was supported by Egger’s test (*P* = .759; Figure 6).

**Figure 6.**
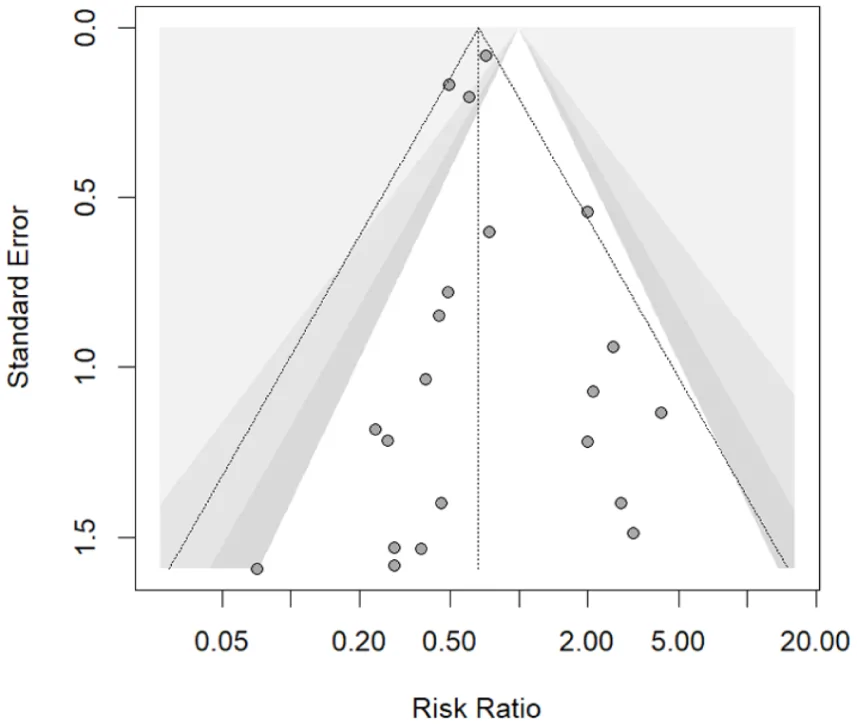
Funnel of stroke studies.

### Paraplegia/Paraparesis/Spinal Cord Ischemia (SCI)

Most meta-analyses found no significant difference between TEVAR and OSR. Donghai Liu et al,^17^ Li et al,^19^ and Jianping Liu et al,^24^ all reported non-significant pooled ORs, with no heterogeneity, indicating similar rates of these neurological complications across interventions.^17,19,24^ Ying Yu et al^25^ also found no difference in paraplegia risk between TEVAR and OSR. The retrospective study by Zhu et al^21^ observed no cases of paraplegia following TEVAR, possibly due to anatomical considerations in graft placement. Meta-analysis of comparative studies from the included SRs demonstrated no significant difference in the risk of paraplegia/paraparesis between TEVAR and OSR (RR: 0.88; 95% CI: 0.53-1.47; *I*^2^ = 22.7%; Figure 7). The funnel plot indicated no evidence of publication bias, supported by Egger’s test (*P* = .894; Figure 8).

**Figure 7.**
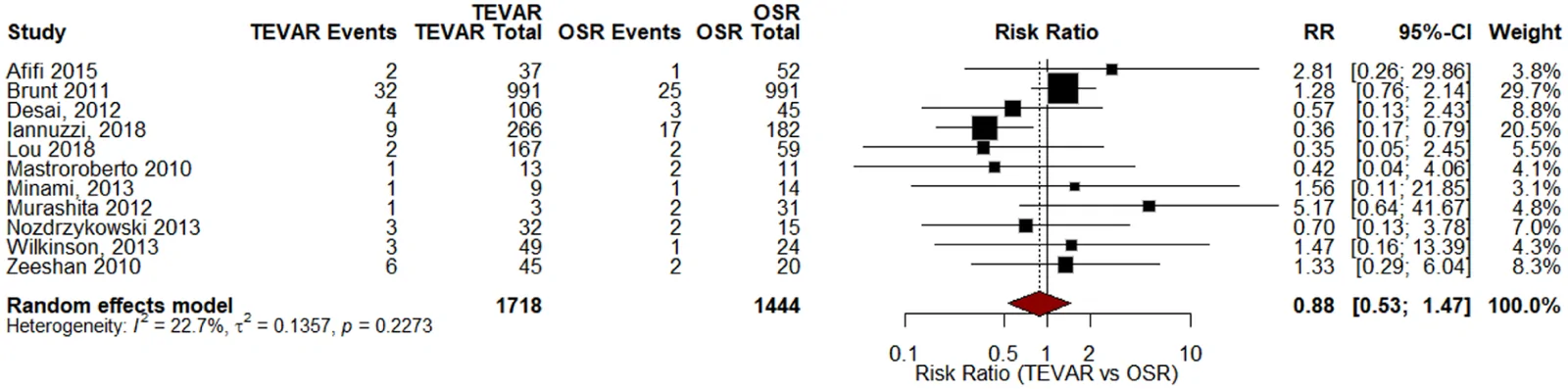
Forest plot for paraplegia studies.

**Figure 8.**
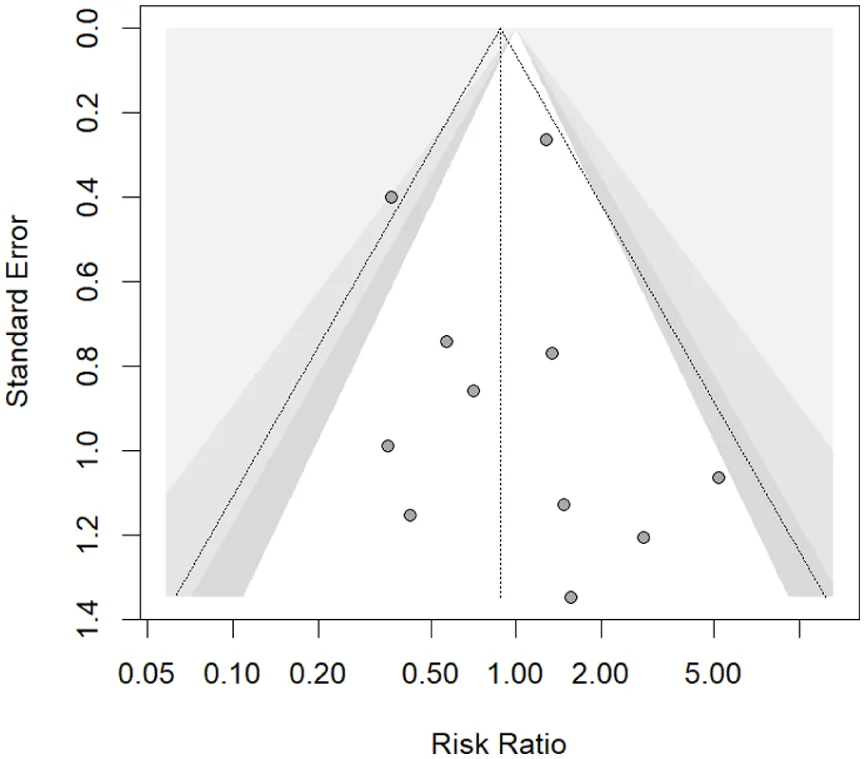
Funnel plot for paraplegia. Egger’s test (*P* = .894).

Boufi et al,^18^ focusing on chronic TBAD, found a higher SCI risk with OSR (OR: 3.3; 95% CI: 0.97-11.25; *P* = .05), with pooled rates of 5% for OSR and 2.2% for TEVAR. Moulakakis et al^22^ reported similar SCI rates between groups, though they noted a generally higher frequency of cerebrovascular and spinal events in OSR. Meta-analysis conducted using the original studies showed a similar result with no significant difference between the 2 approaches (RR: 0.70 95% CI 0.13-3.81; *I*^2^ = 0%; Figure B4).

### Reintervention

Several studies have compared reintervention rates between TEVAR and OSR, with mixed results (Table A3). Ahmed et al^16^ found OSR had the lowest mean absolute risk (0.072 ± 0.027) and the highest probability (97.8%) of being the most favorable option when comparing reintervention. In chronic dissection, Boufi et al^18^ reported significantly higher reintervention with TEVAR (OR: 0.34; 95% CI: 0.16-0.69; *P* = .003), while Harky et al^15^ also observed higher 1-year reintervention rates in TEVAR (15.4% vs 5.5%; *P* = .004). However, other meta-analyses by Jianping Liu et al,^24^ Donghai Liu,^17^ and Li et al^19^ found no statistically significant difference. Lombardi et al^20^ observed follow-up reinterventions, particularly in patients undergoing TEVAR for aberrant anatomy, often due to endoleaks or limb ischemia. However, direct statistical comparisons between TEVAR and OSR were not provided. Overall, while some studies indicated a higher risk with TEVAR, evidence remained inconsistent. A minority of original studies reported Kaplan-Meier curves for reintervention-free survival directly comparing TEVAR and OSR and consistently demonstrating higher reintervention rates after TEVAR.^26-31^ The main indications for reintervention were type I and II endoleaks, persistent false lumen perfusion with aneurysmal degeneration, and, less commonly, stent graft migration or fracture. Aortic remodeling was reported in several series and was generally more favorable after TEVAR than OSR, although definitions and methods varied.^30-33^

### Rupture of TBAD

Evidence on the risk of rupture between TEVAR and OSR in TBAD was mixed. Donghai Liu et al^17^ found no significant difference in rupture incidence between the 2 (OR: 0.61; 95% CI: 0.24-1.56; *P* = .30), supported by Zhu et al^21^ who reported similar rates (TEVAR: 10.9% vs OSR: 11.1%; *P* = .980) in their original work. In contrast, Ahmed et al^16^ reported that OSR had the lowest mean absolute risk for rupture (0.069 ± 0.056) and the highest probability (46.8%) of being the most favorable option overall in reducing the risk of rupture. Their stratified analysis showed that OSR had the lowest rupture risk in both acute TBAD (mean: 0.021 ± 0.028; 86.7% probability) and chronic TBAD (mean: 0.047 ± 0.074; 63.4% probability), although the contrast plot revealed no statistically significant difference between treatments.

Boufi et al,^18^ using non-comparative data, found lower rupture rates with OSR (1.2%) than TEVAR (3%) in chronic cases (data were not compared using a statistical test, pooled separately for the 2 groups, and collected from different studies and therefore, *P* value was not reported), though they were unable to perform a comparative meta-analysis due to limited OSR data and cautioned that rupture after OSR may be underreported.

### Bleeding

TEVAR seemed to be associated with a significantly lower risk of bleeding compared with OSR. Jianping Liu et al^24^ reported a reduced bleeding risk in the TEVAR group (OR: 0.24; 95% CI: 0.14-0.42; *P* < .001) with no heterogeneity (*I*^2^ = 0%) in their meta-analysis. Ying Yu et al^25^ similarly found a lower bleeding risk with TEVAR (RR: 0.44; 95% CI: 0.23-0.83; *P* = .01; *I*^2^ = 56%), and this result was strengthened in a sensitivity analysis (RR: 0.29; *I*^2^ = 0; *P* < .0001). Donghai Liu et al^17^ assessed bleeding among early complications, their pooled results did not report bleeding as a distinct outcome, and their broader conclusions suggested no significant differences in “other complications” between TEVAR and OSR. Nonetheless, the more focused analyses by Jianping Liu et al^24^ and Ying Yu et al^25^ provided consistent evidence supporting a bleeding-related advantage for TEVAR.

### Hospital Stay

Length of hospital stay is consistently reported to be shorter in patients undergoing TEVAR compared with OSR for TBAD. Harky et al^15^ found significantly lower total hospital stay for TEVAR (mean: 7 ± 3.1 days) versus OSR (mean: 15 ± 7.2 days; *P* < .00001), and shorter intensive care unit (ICU) stay (4 ± 2.1 vs 6 ± 3.4 days; *P* = .002). Donghai Liu et al^17^ reported a pooled mean difference of −6.44 days (95% CI: −10.21 to −2.67; *P* = .0008), and Ying Yu et al^25^ similarly found a shorter hospital stay in the TEVAR group standard mean deviation (SMD = −0.84; 95% CI: −1.30 to −0.38; *P* = .0003), confirmed via sensitivity analysis. This advantage is often attributed to TEVAR’s minimally invasive nature. Li et al^19^ also implied prolonged hospitalization for OSR in the context of cost-effectiveness. However, Boufi et al^18^ and Lombardi et al^20^ noted insufficient data for robust comparisons in some studies, and Chandra et al^23^ reported a pooled median stay of 12.5 days for TBAD with mesenteric malperfusion, without distinguishing between interventions.

### Methodological Quality

The methodological quality of the included SRs was assessed using the AMSTAR-2 tool. Of the 11 reviews evaluated, 10 were rated as having low or critically low overall confidence due to 1 or more critical weaknesses (Table 2). In several cases, protocol registration was rated as “Partial Yes” and treated as a weakness, in line with the approach described by De Santis et al,^34^ given its classification as a critical domain. Only 1 review^25^ achieved a moderate confidence rating, with no identified critical flaws. These findings highlight methodological limitations that should be considered when interpreting the review conclusions. Meta-analysis was rated as very low, primarily due to the risk of biases arising from observational studies, inconsistency in the results and imprecision (Table A4).

**Table 2.** Methodological Quality of the Systematic Reviews (AMSTAR-2).

Items		Type	Ahmed et al (2024)	Donghai Liu et al (2020)	Li et al (2018)	Boufi et al (2019)	Lombardi et al (2023)	Zhu et al (2016)	Moulakakis et al (2014)	Chandra et al (2022)	Harky et al (2019)	Jianping Liu et al (2019)	Yu et al (2023)
1)	Did the research questions and inclusion criteria for the review include the components of PICO?		Yes	Yes	Yes	Yes	Yes	Yes	Yes	Yes	Yes	Yes	Yes
2)	Did the report of the review contain an explicit statement that the review methods were established prior to the conduct of the review and did the report justify any significant deviations from the protocol?		Pyes	Pyes	Pyes	Pyes	Yes	Pyes	No	Pyes	Pyes	Pyes	Yes
3)	Did the review authors explain their selection of the study designs for inclusion in the review?		No	No	Yes	Yes	No	Yes	No	No	Yes	No	No
4)	Did the review authors use a comprehensive literature search strategy?		Yes	Yes	Yes	Pyes	Yes	Yes	Yes	Pyes	Yes	Yes	Yes
5)	Did the review authors perform study selection in duplicate?		Yes	Yes	Yes	Yes	Yes	Yes	No	Yes	Yes	Yes	Yes
6)	Did the review authors perform data extraction in duplicate?		Yes	Yes	Yes	No	Yes	Yes	Yes	No	Yes	Yes	Yes
7)	Did the review authors provide a list of excluded studies and justify the exclusions?		Yes	Pyes	Yes	Yes	Yes	No	Yes	Yes	Pyes	Yes	Yes
8)	Did the review authors describe the included studies in adequate detail?		Yes	Yes	No	Yes	Yes	No	No	Pyes	Yes	Yes	Yes
9)	Did the review authors use a satisfactory technique for assessing the RoB individual studies that	RCT	Yes	N/A	N/A	No	No	N/A	No	N/A	N/A	N/A	Yes
		NRSI	Yes	Yes	Pyes	No	No	No	No	No	No	Pyes	N/A
10)	Did the review authors report on the sources of funding for the studies included in the review?		No	No	No	No	No	No	No	No	No	No	No
11)	If a meta-analysis was performed, did the review authors use appropriate methods for statistical combination of	RCT	Yes	N/A	N/A	No	N/A	N/A	No	N/A	N/A	N/A	Yes
		NRSI	Yes	Yes	No	No		No			No	No	N/A
12)	If a meta-analysis was performed, did the review authors assess the potential impact of RoB in individual studies on the results of the meta-analysis or other evidence synthesis?		Yes	Yes	No	Yes	N/A	Yes	No	N/A	No	No	Yes
13)	Did the review authors account for RoB in individual studies when interpreting/discussing the results of the review?		Yes	Yes	No	Yes	No	Yes	No	No	No	No	Yes
14)	Did the review authors provide a satisfactory explanation for, and discussion of, any heterogeneity observed in the results of the review?		Yes	Yes	Yes	Yes	No	Yes	Yes	No	Yes	Yes	Yes
15)	If the review authors performed quantitative synthesis, did they carry out an adequate investigation of publication bias (small study bias) and discuss its likely impact on the results of the review?		No	Yes	Yes	Yes	N/A	No	No	N/A	No	Yes	Yes
16)	Did the review authors report any potential sources of conflict of interest, including any funding they received for conducting the review?		Yes	Yes	Yes	No	Yes	No	Yes	Yes	Yes	Yes	Yes
Number of critical weakness			2	1	2	2	2	4	4	2	4	2	0
Overall confidence rating			C Low	Low	C Low	C Low	C Low	C Low	C Low	C Low	C Low	C Low	Moderate

Abbreviations: C Low, critically low, Pyes, Partial yes; N/A, not applicable; RCT, randomized controlled trial; NRSI, Non-Randomized Studies of Interventions; PICO, Population, Intervention, Comparator, Outcome; RoB, risk of bias.

## Discussion

This is the first UR comparing TEVAR and OSR for TBAD. This UR and meta-analysis provides a high-level synthesis of existing evidence by consolidating 11 systematic reviews—9 of which were eligible for meta-analysis—published over the past decade into the highest tier of evidence currently available. Most notably, our pooled analysis of over 54 000 patients for early mortality demonstrated a clinically significant advantage for TEVAR over OSR. Additionally, this review highlights consistent findings across the literature showing that TEVAR is associated with shorter hospital stays and fewer postoperative complications. Long-term outcomes remain inconclusive.

These findings underscore the need to contextualize current clinical decision-making strategies for TBAD. The management of TBAD requires a tailored approach based on patient risk factors, anatomical considerations, and disease progression. Traditionally, treatment strategies include medical therapy, TEVAR, and OSR. While TEVAR has become the preferred modality in many cases due to its minimally invasive nature, OSR and optimal medical management remain essential options for select patients.^35^

TEVAR has revolutionized the management of TBAD, offering a less invasive alternative to OSR with improved recovery times and reduced morbidity. It is primarily indicated in complicated acute TBAD, where patients present with life-threatening conditions such as aortic rupture, malperfusion syndromes, or rapid expansion of the false lumen.^35^ Studies have demonstrated that TEVAR effectively restores true lumen patency and prevents further dissection-related complications.^36-38^ Additionally, TEVAR is recommended for high-risk patients who may not tolerate OSR due to advanced age, multiple comorbidities, or frailty. The procedure’s lower perioperative mortality and morbidity make it a preferred choice in these scenarios.^39^ However, successful TEVAR depends on favorable anatomical conditions, particularly adequate proximal and distal landing zones for stent-graft deployment.^36,37^

Despite the growing preference for TEVAR, OSR remains a crucial intervention for TBAD in specific situations. It is primarily indicated in patients with unfavorable aortic anatomy, such as inadequate landing zones, excessive vessel tortuosity, or significant aortic branch involvement that would preclude a successful endovascular repair. Additionally, chronic TBAD with complications, such as progressive aneurysmal degeneration, persistent false lumen perfusion, or aortic rupture, often necessitates OSR. While OSR is associated with higher perioperative risks and longer recovery times, it provides a durable long-term solution in cases where TEVAR is not feasible or has failed. Surgical expertise, patient selection, and postoperative care are critical in determining outcomes following OSR.^40-43^

Our findings emphasize the significant advantages of TEVAR over OSR in managing TBAD. TEVAR was associated with notably lower odds of early mortality. These findings support previous research that highlighted TEVAR’s role in improving short-term survival rates. Furthermore, patients undergoing TEVAR experienced fewer cardiac and pulmonary complications and benefited from shorter hospital stays, underscoring its less invasive and safer nature for early recovery.^17^

Further research using decision-analytic models has provided insights into the advantages of TEVAR across diverse age groups and clinical contexts. A prior report showed ~8.5 additional months of perfect health in patients undergoing TEVAR, with perioperative mortality emerging as the key factor driving this outcome.^44^

Despite these promising short-term results, the long-term survival benefits of TEVAR remain a subject of debate. A single-center retrospective study and meta-analysis reported a 10-year overall survival rate of 41.9%, with no statistically significant difference between the OSR (56.7%) and TEVAR (26.1%) groups (log-rank *P* = .953). Factors such as refractory hypertension and a preoperative aortic diameter exceeding 55 mm were identified as critical predictors of long-term outcomes.^21^

Our results showed that TEVAR has a lower risk of postoperative stroke but not for paraplegia/paraparesis or SCI compared with OSR. Yet the review of systematic reviews showed that TEVAR consistently demonstrates lower risks of cardiac, pulmonary, renal, and bleeding complications, as well as shorter hospital stays. However, no significant difference was observed in paraplegia or spinal cord injury, and findings on stroke, rupture, and reintervention remain mixed across most systematic reviews, reflecting variability in patient populations, follow-up durations, and outcome definitions.

Spinal cord ischemia, leading to paraparesis or paraplegia, can occur following the repair of AAA and varies depending on the type of repair and the extent of the aortic pathology. This is likely precipitated by perioperative hypotensive events, disruption of blood flow in intercostal and lumbar arteries, and/or thromboembolic events in costo-lumbar arteries.^25^ Additionally, disturbances in the extrinsic pathway blood supply to the anterior spinal artery, particularly through the artery of Adamkiewicz, play a significant role.^20^

There are conflicting findings regarding whether different techniques influence the risk of neuroendovascular complications.^15^ A retrospective study with 6202 patients reported no significant difference between TEVAR and OSR in the incidence of postoperative paraplegia.^45^ While the traditional surgical approach involves cross-clamping of the aorta, increasing the risk of SCI, vigorous aortic manipulation in TEVAR can disrupt the intercostal collateral network, leading to ischemia.^46^

Reducing the overall risk of SCI is crucial. The mechanism behind this complication is multifactorial, including the extent of aortic repair, the length of aortic and collateral circulatory coverage, embolization, and diminished spinal cord perfusion pressure (SCPP). Adjunct strategies to mitigate SCI risk include cerebrospinal fluid drainage (CSFD), revascularization of the left subclavian artery (LSA), permissive hypertension, and perioperative steroid and naloxone use.^47^

Zheng et al conducted a systematic review of only TBAD patients who underwent TEVAR, revealing that prophylactic CSFD, compared with the non-prophylactic group, was not associated with a lower rate of SCI incidence or a reduction in 30-day or in-hospital mortality rates.^48^ Another meta-analysis showed that CSFD use in both OSR and TEVAR groups had a complication rate of 6.5% and a 0.9% associated mortality rate.^49^

The left subclavian artery (LSA) coverage, as well as the hypogastric artery coverage, interrupts collateral circulation to the spinal cord, causing ischemic complications.^40^ Therefore, revascularization is essential to reduce the risk of SCI, limb ischemia, vertebrobasilar ischemia, and maintain long-term patency.^50-53^

The concept of permissive hypertension, which is typically used with CSFD in high-risk patients, aims to maintain the perioperative mean arterial pressure at >80 mmHg, with a possible increase if necessary. This measure augments the spinal cord perfusion pressure (to protect the spinal cord, as perioperative hypotension should be avoided.^50,54^

Stroke is another widely reported postoperative neurological complication among both techniques, which can result in permanent neurologic deficits in most cases. It is due to the spread of atherosclerotic and cellular debris post-treatment in patients older than 65 years or those susceptible to forming atheromas. Similarly, pulmonary veins are susceptible to embolic dissemination, resulting in lung damage after surgery.^16^ Cardiac complications, such as myocardial infarction, occur due to the burden major surgery creates, triggering thrombogenicity, and circulating tissue factors. Additionally, increased sympathetic activity could cause a mismatch between the oxygen supply and demand of the heart, potentially leading to MI within 24 to 72 hours.^55^

Inadequate perfusion of renal arteries during surgical management can cause renal dysfunction. This was previously regarded as a risk of TEVAR, as the stent elicits an inflammatory response alongside the use of contrast.^16^ However, advancements in stent technology have lessened this concern, making TEVAR a safer option.^56^ There is still room to further improve the efficacy of endovascular interventions, as the risk of major complications with lasting damage remains present even with the TEVAR approach. Other complications of TEVAR include endoleaks, stent fractures, and aortic remodeling.^57^ Endoleaks (blood into the aneurysm sac), are the most common complications, include 5 types in which types I and III require urgent intervention. Stent fractures (tears or disruptions) can occur and lead to endoleak type III requiring repair. Aortic remodeling (such as elongation or curviness after EVAR) may necessitate close observation and assessment.^58^ Advances in the TEVAR devices may target measures to overcome these complications.^59^

### Limitations

This review has several limitations stemming from the umbrella-level synthesis and the subsequent meta-analyses conducted using primary studies. At the umbrella review level, a key limitation lies in the duplication of evidence—many primary studies were included in multiple SRs, which may have led to double-counting of studies and patients. This redundancy introduces the potential for overrepresentation of certain findings. Additionally, the heterogeneity in scope, quality, and inclusion criteria across SRs limited the ability to directly pool summary estimates from each SR. Variations in outcome definitions, follow-up durations, patient populations (acute vs chronic TBAD), and analytic methods (eg, ORs vs RRs) further restricted the comparability between reviews. As such, the umbrella synthesis was largely qualitative, aimed at identifying consistencies and gaps rather than generating pooled effect sizes from SRs. Moreover, one of the limitations is procedural heterogeneity, such as the stent graft used, the use of left subclavian artery coverage or revascularization and the perioperative cerebrospinal fluid drainage protocol for reducing the risk of spinal cord ischemia and its impact on the overall outcomes.

At the meta-analysis stage, although duplicate patient data were minimized by selecting only unique primary studies, many studies were retrospective observational cohorts, susceptible to selection bias and confounding. Moreover, there was substantial heterogeneity across studies in terms of baseline characteristics, surgical techniques, and outcome reporting. Most TEVAR and OSR series involved complicated TBAD cases (eg, rupture, malperfusion, refractory pain, or aneurysmal degeneration), whereas uncomplicated TBAD is more often managed medically. Only a small number of studies clearly separated and analyzed uncomplicated cohorts, limiting subgroup comparisons. This was reflected in the *I*^2^ values and τ^2^ estimates for several outcomes, particularly in long-term survival and complication rates. We attempted to mitigate this using random-effects models, but unexplained heterogeneity still limits the certainty of pooled estimates. For certain outcomes (such as SCI), the number of comparative studies was limited, reducing statistical power. There is a possibility of an overlap in primary studies across multiple systematic reviews, however, there is currently no standard methodological approach to deal with overlapping in primary studies across reviews^60^ and we meticulously addressed this point to ensure no overlap in the studies included in the meta-analysis. Also, the time lag could be a limitation, since older reviews might not reflect current practices and standards. Recent advances in TEVAR devices and newer technology could further reduce complications and impact decision-making in the management of TBAD.^61^ Therefore, our analysis extended until the current year. The analysis did not consider the recent TEM classification (Type-Entry-Malperfusion classification) as there are not enough studies or SR to consider and therefore, we focused on type B.^62^ Finally, publication bias was suggested in some analyses (eg, short-term mortality), as evidenced by funnel plot asymmetry and a significant Egger’s test. While we conducted additional sensitivity analyses, the number of studies removed for this analysis were substantial.

Despite these limitations, this review offers the most current and comprehensive synthesis of available evidence comparing TEVAR and OSR in TBAD and should serve as a useful reference point for future research and clinical decision-making.

## Conclusion

This UR highlights the advantages of TEVAR over OSR in the management of TBAD, particularly in terms of early mortality, recovery, and postoperative complications. However, long-term outcomes between the 2 approaches remain similar, with no significant difference in survival rates. Future research, including RCTs and long-term studies, is necessary to better define the role of TEVAR in the treatment of TBAD. The management of TBAD should be individualized based on patient characteristics, anatomical considerations, and the severity of the dissection, with careful selection of treatment strategies to minimize risks and improve outcomes.

## Supplemental Material

sj-docx-1-ang-10.1177_00033197251392660 – Supplemental material for Open Surgical Versus Endovascular Repair for Type B Aortic Dissection: Umbrella Review and Meta-Analysis Supplemental material, sj-docx-1-ang-10.1177_00033197251392660 for Open Surgical Versus Endovascular Repair for Type B Aortic Dissection: Umbrella Review and Meta-Analysis by Hassan Al-Thani, Osamah Alrawi, Eman Elmenyar, Mashhood Naduvilekandy, Shams O. Alkhateeb, Waqar Mogassabi, Lama Alkahlout and Ayman El-Menyar in Angiology

## Figures and Tables

**Figure B1.**
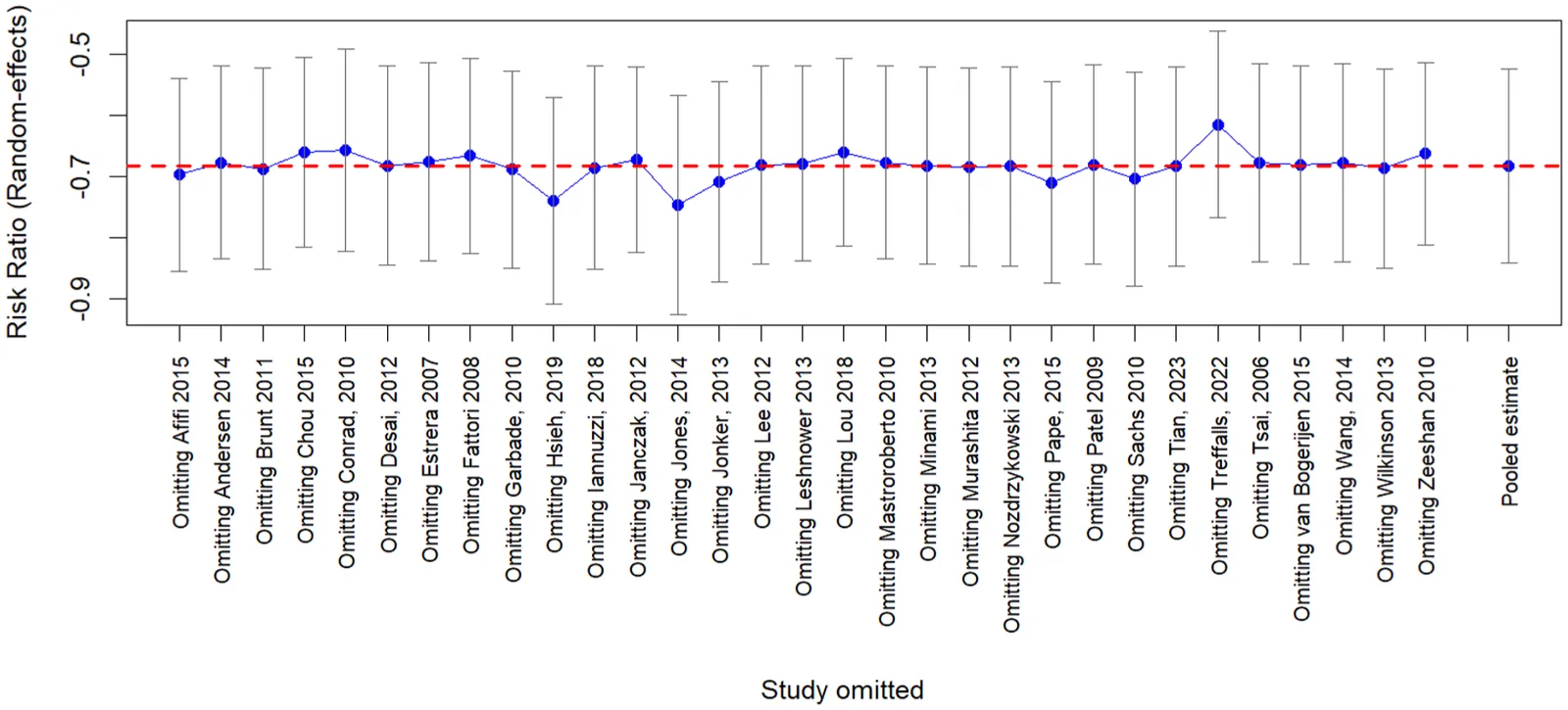
Forest plot and line diagram for leave one out sensitivity analysis of early mortality.

**Figure B2.**
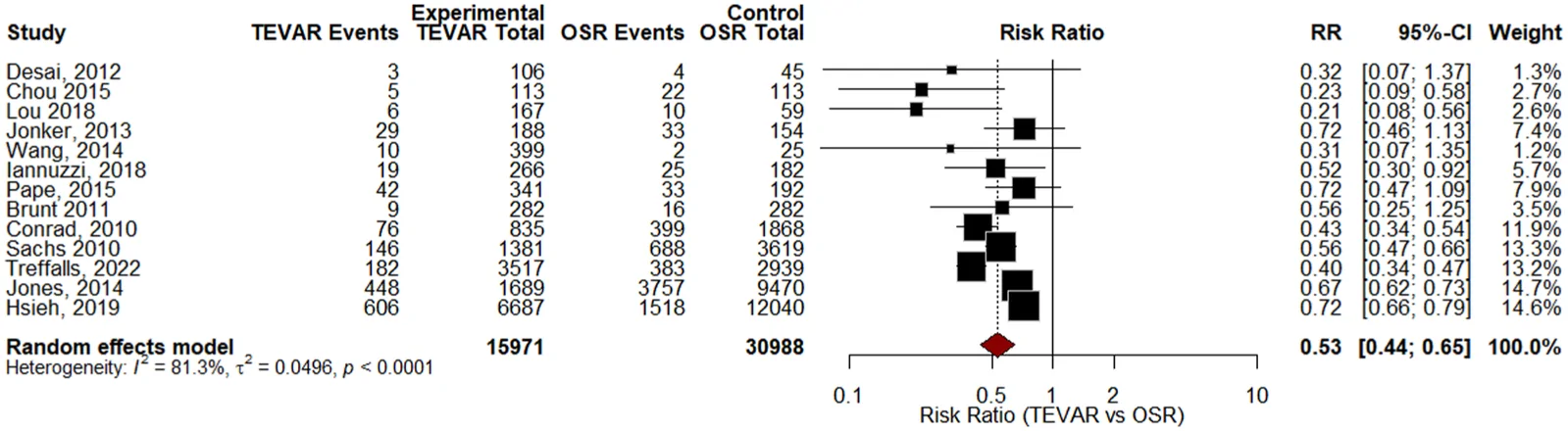
Forest plot for short term mortality (sensitivity analysis, excluding total patients <150).

**Figure B3.**
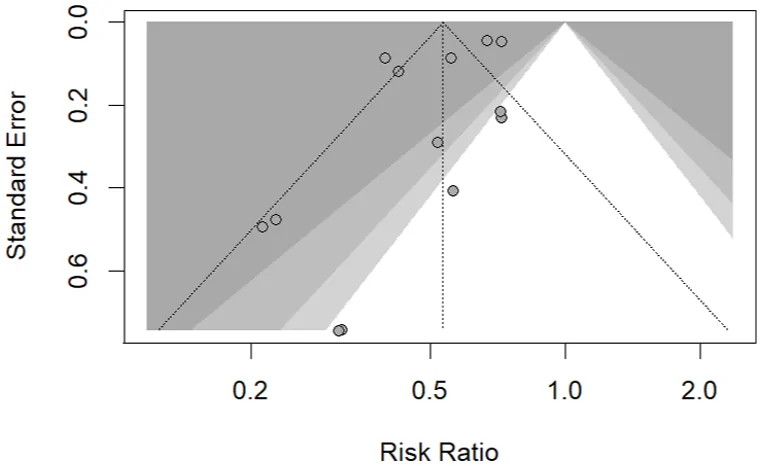
Funnel plot for short term mortality sensitivity analysis Egger’s test (*P* > .05).

**Figure B4.**
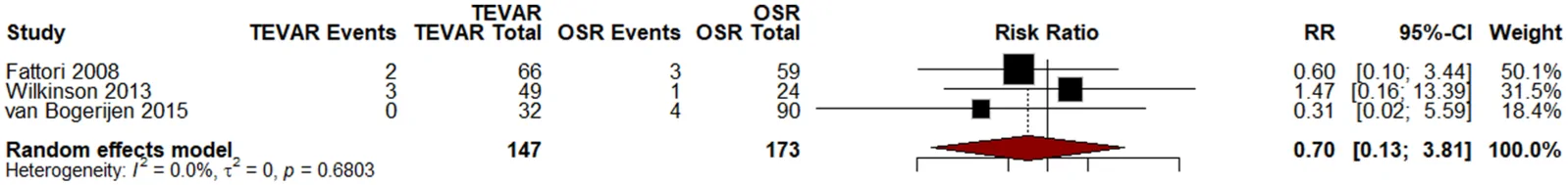
Forest plot for SCI.

## Appendix A

**Table A1.** Short-Term Mortality Retrieved From the SRs.

Study	TEVAR	OSR	Effect estimate (95% CI or as reported)
Harky et al^15^	7.4%	18.6%	*P* < .0001 (no OR reported)
Ahmed et al^16^	Mean AR: 0.08 ± 0.014	Mean AR: 0.202 ± 0.026	Significant difference between OSR and TEVAR; (ORs not explicitly reported)
Donghai Liu et al^17^	9.1%	14.7%	OR: 0.54 (0.43-0.68)
Boufi et al^18^	2% (Non-comparative pooled)	9.3% (Non-comparative pooled)	OR: 0.24 (0.06-0.91)
Li et al^19^	Not specified	Not specified	OR: 0.49 (0.29-0.81)
Lombardi et al^20^	2.7%	4.5%	*P* = NS
Moulakakis et al^22^	7.3%^a^	19.0%^a^	NA
Chandra et al^23^	13.44%	11.44%	*P* = .762 (no OR reported)
Liu et al^24^	Not specified	Not specified	OR: 0.40 (0.28-0.56)
Ying Yu et al^25^	Not specified	Not specified	RR: 0.59 (0.49-0.73)

Rates are given in percentage, unless specified explicitly.

Abbreviations: NS, not significant; AR, absolute risk; OR, odds ratio; RR, risk ratio; SRs, systematic reviews; CI, confidence interval (SRs).

a Complicated TBAD cases.

**Table A2.** Complications Retrieved From the SRs.

References	HLOS		Cardiac		Pulmonary		Renal		Stroke	
	OSR	TEVAR	OSR	TEVAR	OSR	TEVAR	OSR	TEVAR	OSR	TEVAR
Harky et al^15^	15 ± 7.2	7 ± 3.1	NA	NA	NA	NA	NA	NA	NA	NA
Ahmed et al^16^	NA	NA	MAR = 0.184 ± 0.051	MAR = 0.184 ± 0.051	MAR = 0.34 ± 0.076	MAR = 0.282 ± 0.06	RF: MAR = 0.2 ± 0.027	RF: MAR = 0.119 ± 0.022	MAR = 0.066 ± 0.021	MAR = 0.038 ± 0.009
Liu et al^17^	TEVAR vs OSR MD −6.44 (−10.21 to −2.67)		OR: 0.42 (0.33-0.54)		OR: 0.57 (0.48-0.68)		RF OR: 0.57 (0.30-1.06)		OR: 1.11 (0.42-2.89)	
Boufi et al^18^	NA	NA	NA	NA	24.90%	4%	Dialysis: 5.2%	Dialysis: 0.8%	4.50%	2.70%
Li et al^19^	NA	NA	NA	NA	NA	NA	RF OR = 0.63 (0.32-1.24)		OR = 1.10 (0.53-2.28)	
Moulakakis et al^22^	NA	NA	NA	NA	NA	NA	NA	NA	6.80%	3.90%
Chandra et al^23^	12.5 d (IQR = 8-21 d)		NA	NA	NA	NA	NA	NA	NA	NA
Liu et al^24^	NA	NA	MI OR: 0.79 (0.29-2.16)		OR: 0.51 (0.32-0.82)		OR: 0.53 (95% CI: 0.30-0.94)		OR: 0.98 (0.48-1.97)	
Yu et al^25^	SMD = −0.84 (−1.30 to −0.38)		NA	NA	RR: 0.67 (0.60-0.76)		Dialysis RR: 0.55 (0.47-0.65)		RR: 0.71 (0.51-0.98	

OR and RR is given as TEVAR vs OSR unless specified.

Abbreviations: HLOS, hospital length of stay; MAR, mean absolute risk; RF, renal failure; SMD, standard mean difference; NA, not available; MI, myocardial Infarction; TEVAR, thoracic endovascular aortic repair; OSR, open surgical repair; IQR, interquartile range; OR, odds ratio; RR, risk ratio; SRs, systematic reviews.

**Table A3.** Complications Retrieved From the Included SRs (Continued).

References	Paraplegia/Paraparesis		Rupture		Reintervention		SCI	
	OSR	TEVAR	OSR	TEVAR	OSR	TEVAR	OSR	TEVAR
Harky et al^15^	2.90%	1.30%	NA	NA	NA	NA	NA	NA
Ahmed et al^16^	MAR = 0.074 ± 0.031	MAR = 0.05 ± 0.019	MAR = 0.069 ± 0.056	MAR = 0.069 ± 0.037	MAR = 0.072 ± 0.027	MAR = 0.172 ± 0.039	NA	NA
Liu et al^17^	OR: 1.19 (0.73, 1.94)		OR: 0.61 (0.24, 1.56)		NA	NA	NA	NA
Boufi et al^18^	NA	NA	1.20%	3%	11.80%	20.20%	5%	2.20%
Li et al^19^	OR = 1.27 (0.7902.04)		NA	NA	Late re-intervention: OR = 1.23 (0.55-2.75)		NA	NA
Lombardi et al^20^	NA	NA	NA	NA	13.60%	5.20%	NA	NA
Moulakakis et al^22^	NA	NA	NA	NA	NA	NA	3.3.%	3.10%
Liu et al^24^	OR: 0.71 (0.31-1.61)		NA	NA	OR: 2.94 (0.81-10.71)		OR: 0.69 (0.19-2.49)	
Yu et al^25^	No significant risk		NA	NA	NA	NA	NA	NA

OR and RR is given as TEVAR vs OSR unless specified.

Abbreviations: MAR, mean absolute risk; NA, not available; TEVAR, thoracic endovascular aortic repair; OSR, open surgical repair; OR, odds ratio; RR, risk ratio; SRs, systematic reviews.

**Table A4.** GRADE Analysis for Outcomes in Meta-Analysis.

Outcome	Limitations	Upgrade	GRADE rating	Effect estimate (RR)
Short-term mortality	Risk of bias, inconsistency, publication bias	None	⊕ ○○○ Very low	0.51 (0.43-0.59)
Stroke	Risk of bias, inconsistency	None	⊕ ○○○ Very low	0.67 (0.54-0.82)
Paraplegia	Risk of bias, imprecision	None	⊕ ○○○ Very low	0.88 (0.53-1.47)

High quality observational studies can be upgraded (given positive scores) based on some criteria rather than downgrading from starting score. The symbol represents the score, 4 ⊕ symbol would mean very high rating, and 1 means very low.
